# Supplementary material for: Inequality is rising where social network segregation interacts with urban topology
Source: Nat Commun. 2021 Feb 18;12:1143. doi: 10.1038/s41467-021-21465-0 (PMC7892860; doi:10.1038/s41467-021-21465-0)
Supplement: Supplementary file 1 — Supplementary Information [file 41467_2021_21465_MOESM1_ESM.pdf]

Supplementary Information for Inequality is rising where social network segregation interacts with urban topology

Gergő Tóth*[a,b], Johannes Wachs*[c,d], Riccardo Di Clemente[e,f], Ákos Jakobi[g,h], Bence Ságvári[a,i,k], János Kertész[j], and Balázs Lengyel†[a,k,l]

[a]Agglomeration and Social Networks Lendület Research Group, Centre for Economic- and Regional Studies, Hungary
[b]Spatial Dynamics Lab, University College Dublin
[c]Institute for Information Business, Vienna University of Economics and Business
[d]Complexity Science Hub Vienna
[e]Department of Computer Science, University of Exeter
[f]Centre for Advanced Spatial Analysis, University College London
[g]Department of Regional Science, Eötvös Loránd University
[h]Institute of Advanced Studies, Kőszeg Hungary
[i]CSS-Recens, Centre for Social Sciences, Hungary
[j]Department of Network and Data Science, Central European University
[k]International Business School Budapest
[l]Institute for Advanced Studies, Budapest Corvinus University

*G.T. and J.W. contributed equally to this work.
†Corresponding author: E-mail: lengyel.balazs@krtk.mta.hu

Supplementary Note 1: Gini coefficient as the measure of local income inequalities

We adopt the Gini index to quantify economic inequality in towns from total income distributions across income bins pre-defined by the Hungarian Statistical Office. This widely used indicator is defined by the following equation:

$$G_{i,t} = \frac{\sum_{p=1}^{n} \sum_{q=1}^{n} \mid x_p - x_q \mid}{2n \sum_{p=1}^{n} x_q} \tag{S1}$$

where i refers to a town, t denotes the year (either 2011 or 2016), x_p and x_q are the sum of total income in income categories p and q, and n denotes the number of income categories within towns.

Supplementary Note 2: Online social network data and representativity

About the data

The iWiW (International Who Is Who) was launched in 2002 and shortly became the most widely used online social network in Hungary. At its peak around 2010, it was one of the most visited national websites reaching the majority of internet users of the country. During the first few years of operation iWiW provided only basic functionalities, mostly built around finding present and former friends, classmates, colleagues, and looking through one's acquaintance's' acquaintances. Later, photo upload, news-feed (similar to Facebook), messaging, applet to visualize connections and the ability to develop external applications was introduced to the service. But all these came too late, so due to the increasing maintenance costs, low profitability and tough competition from Facebook the site was closed down permanently on June 30, 2014. Although the number of daily visitors begun to fall back significantly from 2011-2012, users rarely deleted their profiles: they just abandoned the service.

In February 2013, the entire dataset of iWiW with basic user information (i.e. date of registration, gender, age, etc.) and connection data (establishment of friendship ties) was made available for us for scientific research purposes.

Our illustrations of the networks of two settlements, Ajka and Gödöllő were constructed as follows. We randomly sampled 1500 users from each town that had at least 20 connections to other local residents. We filtered out all connections between individuals with less than five neighbors in common, then sampled at random 400 users in the giant connected component of the remaining graph.

Representativity

IWiW is a large scale dataset available for research regarding the social connections of the Hungarian population. Use of the service was limited to those aged over 14, so theoretically the maximum number of potential users was 8,2 million people in Hungary. The total number of users who chose a Hungarian settlement as their home location reached 2.8 million by early 2013 (another 600.000 users were outside Hungary). This implies that about 33% of Hungarians older than 14 years were part of the network. Considering the level of internet users measured by nationally representative surveys (76%) in 2013, close to 50% of the adult online population were also iWiW users. In our analysis, social connections represented online are used as a proxy of real-life social connections. This approach is certainly a simplification of the complex social reality, but we argue that despite our data is imperfect and has certain limitations (i.e. we do not know about the nature of social connections, their strength, frequency of communication, etc.), until now it is still the best available source, and there is no such systematic bias in the data that would question the validity of the analysis. The latter is demonstrated in Figure 1(A) comparing the number of individuals by age (14 to 80) for the total population of Hungary, its estimated online population, and the number of users registered at iWiW. Until 60 years the representation of iWiW users follows the estimated number of internet users without any serious deviation. (B) The iWiW user/total population ratio reaches its maximum (60%) around the age of 30, and then starts decreasing continuously, and falling below 30 % above 50, and 10% above 70 years. However, the economically active population of Hungary was well represented on the network.

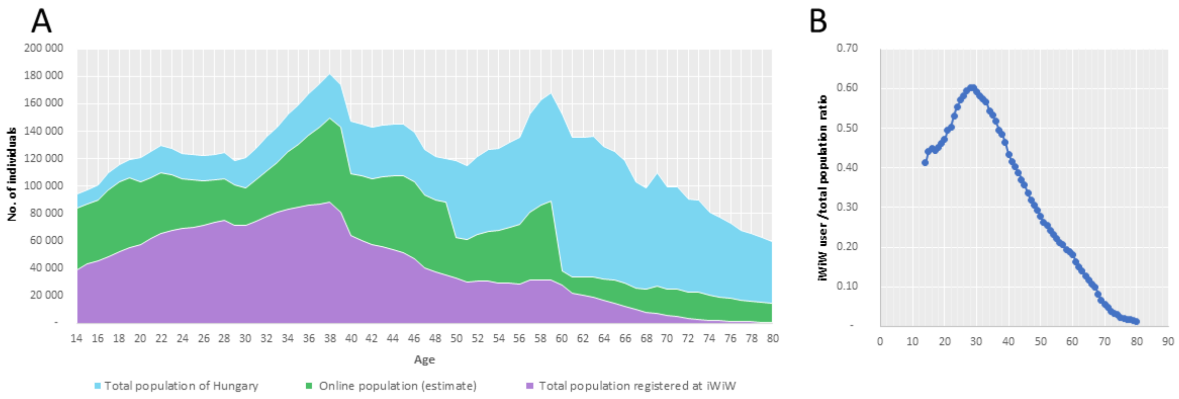

Supplementary Figure 1: **iWiW users compared to the Hungarian population (A)** Comparison of (1) total population of Hungary, (2) estimated online population using nationally representative survey data from 2013, (3) total population registered at iWiW.**(B)** Ratio of iWiW users and total population of Hungary by age.

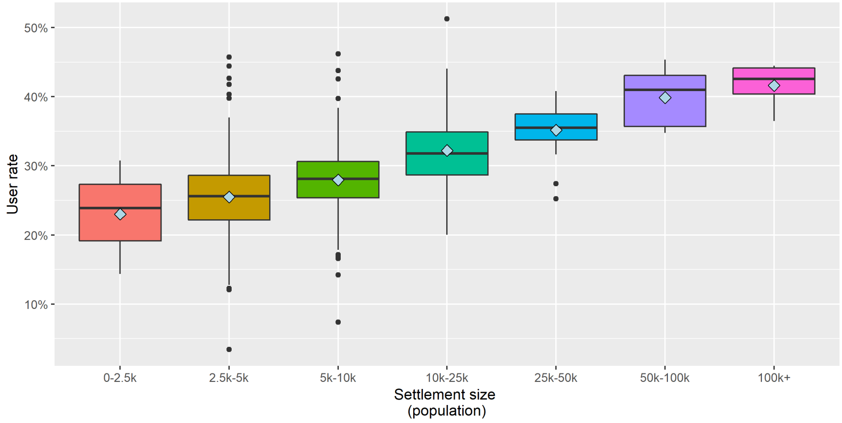

Supplementary Figure 2: iWiW user rate by settlement size categories (n=2557). Lightblue diamonds denote the mean, centres denote the median of distributions. Bounds of boxes are the first and third quartiles and whiskers are defined by 1.5 times the interquartile range. We apply no rules to define minima and maxima.

Potential biases in geographical representativity

Since our analysis is focusing on individual social connections aggregated at settlement level, it is necessary to check for under-representation of certain types of settlements according to their size. The diffusion of innovations - such as the use of an online social network - follows more or less universal patterns, where age, level of education and location play a crucial role. During the fist few years of its life cycle iWiW was mostly used by young, highly educated urban population. Later, more and more elderly people joined from rural areas of the country, however their level of penetration never reached that of the former groups. Figures 2 and 3 illustrate that the overall level of iWiW user rate by settlement size varied between 23% (for small villages) and 42% (for major cities). Since the elderly and individuals with lower educational attainment are over-represented in smaller settlements these figures are not surprising. However, the number of outlier settlements is relatively low, legitimating the use of our data.

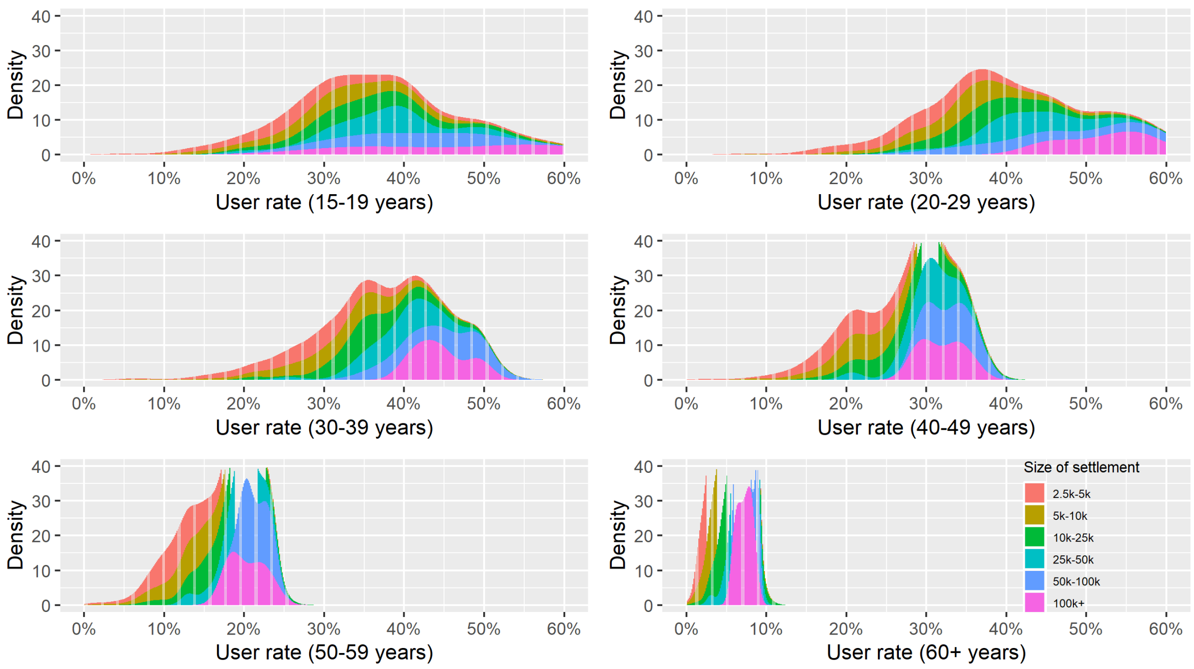

Supplementary Figure 3: Density function of iWiW users in different age groups by size of settlement

Supplementary Note 3: Potential underestimation of fragmentation effect due to low-income under-representation

The local adoption of online social networks, and iWiW in particular, is influenced by the level of economic development in towns. In a previous research, positive correlation was found between income per capita and fraction of iWiW users in town population [1].

In Figure 4, we compare the rate of iWiW users in town population in those towns of our sample where income per capita is higher than the country average (High income) with towns where where income per capita is lower than the country average (Low income). One can observe that iWiW represents town population to a greater extent in rich towns. The t-test reports that the mean of user rate is 7% higher in rich towns and the Kolmogorov-Smirnoff test is also significant confirming that user rate distributions are different across the two sets of towns.

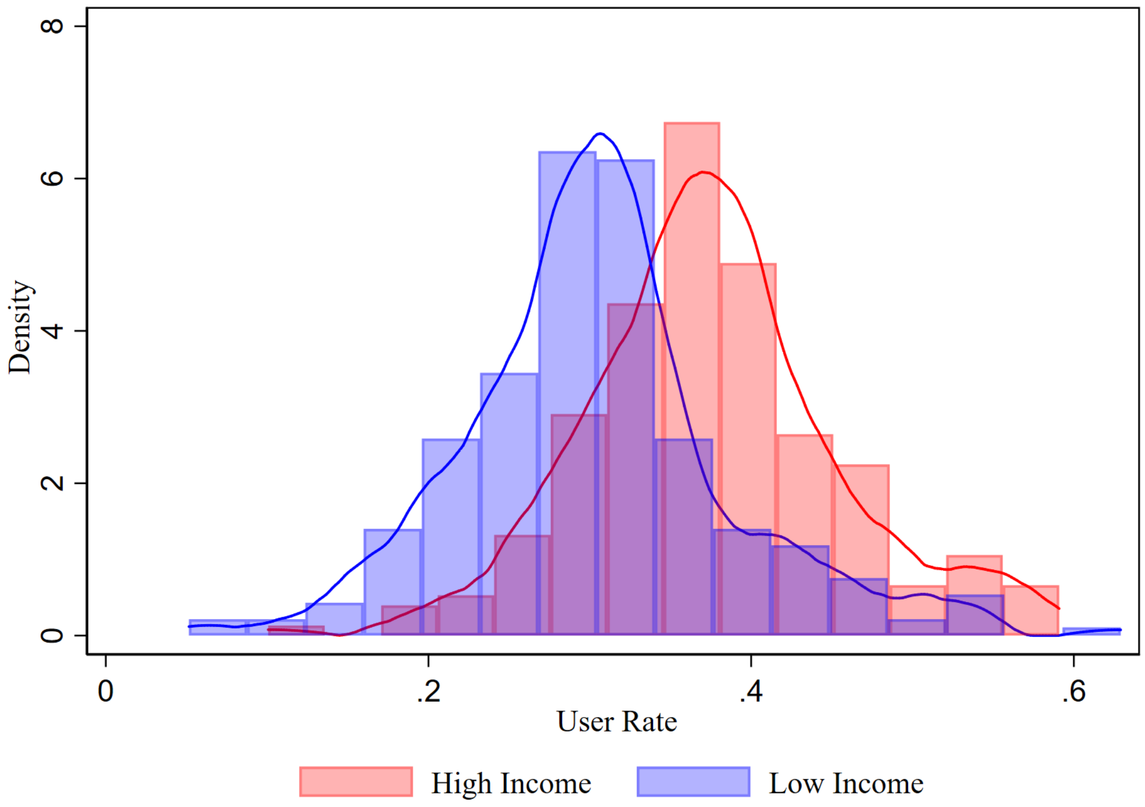

Supplementary Figure 4: The rate of iWiW users in high-income versus low-income towns

These findings are in line with our expectations confirming that low-income individuals might be missing from iWiW. This potentially biases the statistical correlation of social network fragmentation with income inequality. It is, however, plausible to think that those individuals who are not connected to iWiW are segregated from those who are connected to iWiW. Therefore, social network fragmentation in reality might be even stronger in poor towns than what we observe in iWiW. Consequently, the correlation between Fragmentation (F_i) and income inequality (G_i), reported in Figure 1 and in Supplementary Information 4 is most likely underestimated.

Supplementary Note 4: Regression table of estimating income inequality with network fragmentation

Supplementary Table 1: OLS regression with interaction variables. Standard errors in parentheses; all variables have been standardized.

	Dependent variable:	
	$G_{i,2016}$	
	(1)	(2)
F_i	0.051** (0.017, 0.084)	0.085*** (0.048, 0.122)
$G_{i,2011}$	0.858*** (0.822, 0.894)	0.882*** (0.845, 0.919)
Population Density		−87.664 (−177.264, 1.936)
Income per Capita		−0.00000*** (−0.00000, −0.00000)
Userrate		0.384 (−0.078, 0.845)
F_i x $G_{i,2011}$	0.059*** (0.030, 0.087)	0.075*** (0.046, 0.104)
Constant	−0.017 (−0.050, 0.016)	0.243** (0.075, 0.412)
Observations	474	474
R^2	0.824	0.830
Adjusted R^2	0.823	0.828
Note:		*p<0.1; **p<0.05; ***p<0.01

Supplementary Note 5: Principal components of urban topology indicators

To decrease the dimensions of the urban topology approaches taken in the main text, we constructed the *Composite Urban Topology Index* ($CUTI$)from *Average Distance from the Center* (ADC), Segregation by *Physical Barriers* (SPB), and *Spatial Concentration of Amenities* (SCA) by using principal component analysis. Because we cannot argue against reverse causality in the case of SCA, as a robustness check, we constructed the Principal Component from ADC and SPB indices only $PC(ADC, SPB)$. Both of the composite measures have been tested in the remaining empirical analysis and results are reported on both in Supplementary Information 5 and 6. In the main text, we report results from separated analyses only.

Supplementary Table 2: The components of urban topology

	PC(ADC, SPB)	*Composite Urban Topology Index*
Average Distance from the Center (ADC)	0.710	0.612
Segregation by Physical Barriers (SPB)	0.710	0.612
Spatial Concentration of Amenities (SCA)		0.495
Eigenvector value of the first component	1.4	1.60
Variance explained by the first component	.70	.55

Table S2 summarizes information about the principal component analysis. *Composite Urban Topology Index*: 56 percentage of the variance in all three measures is explained by the first component of the principal component analysis using ADC, SPB, and SCA with the eigenvector value of 1.66. *PC(ADC, SPB)*: 70 percentage of the variance in both measures is explained by the first component of the principal component analysis with the eigenvector value of 1.4.

High values of all urban topology approaches refer to high spatial segregation induced by distance (ADC), physical barriers (SPB) and concentration of amenities (SCA). Consequently, high levels of both *PC(ADC, SPB)* and the *CUTI* refers to high spatial segregation in all dimensions included.

Supplementary Note 6: Description of control variables, their distributions, and correlation tests

To evaluate the importance of urban structure in social network fragmentation in towns, we apply a machine learning approach and consider further social and demographic factors that can be sources of social segregation besides urban topology. Description of these variables are as follows:

- *Ethnic fragmentation*: We collected data of population distribution across ethnic groups (hungarian, roma, german etc.) from `https://www.teir.hu` and calculated the entropy of the size distribution of ethnic groups. This indicator is high if ethnic groups in the town have similar sizes. Because link formation is less likely across groups of similar size than between a small group and a large group [?], we expect a positive correlation between the index and social network fragmentation.
- *Religious fragmentation*: We collected data of population distribution across confession groups (catholic, lutheran, muslim etc.) from `https://www.teir.hu` and calculated the entropy of the size distribution. The indicator is high if religious groups in towns have similar sizes. Like in the case of Ethnic fragmentation, we expect a positive correlation between the index and social network fragmentation.
- *Political fragmentation*: We calculate the coefficient of variance in the vote share given to right-wing across voting districts in the town. The indicator is high if voting district differ in terms of political preferences. In our specific country case, there is a large ideological difference between the governing right-wing party and the opponent parties, which might be reflected in everyday social interactions as well. Therefore, we expect a positive correlation between the index and social network fragmentation. Data on parliamentary elections in Hungary was collected directly from the Hungarian National Election Office's official website: `https://www.valasztas.hu/`. Voting outcomes for the different party lists are available at the level of voting precincts.
- *Education inequalities*: We calculate the coefficient of variance in 6th grade math exam. The indicator is high if there is large differences across primary schools in the commuting zone of the town. The quality of schools plays an important role in opportunities for individual progress. Further, school quality differences reflect the divergence of human capital accumulation in the town across generations. Consequently, we expect a positive correlation between this indicator and social network fragmentation. Education data was collected from the national 6th grade competence test in mathematics, that includes individual level data on all primary school students in Hungary in 2011. The raw data is available from the Databank of the Research Centre for Economic and Regional Studies, Hungarian Academy of Sciences. Access can be requested at `http://www.krtk.mta.hu/szervezet/adatbank/`.

Figure S4 illustrates the distribution of the above social segregation variables and our urban topology indicators and their correlation.

In the second stage of the 2SLS regression (Equation 4 in the main text), we include the following control variables that are expected to influence inequalities in towns:

- *Population density*: number of inhabitants divided by the size of the residential area.
- *High school*: the ratio of residents with high school degree or above.
- *Age*: the ratio of residents older than 60 years.
- *Unemployment ratio*: number of unemployed people as a percentage of labour force.
- *Distance to border*: the distance from the nearest border measured in kilometers.
- *Foreign investment*: revenue capital owned by foreign firms, measured in 1000 Hungarian Forint.

All data required to calculate the control variables was retrieved from `https://www.teir.hu`. Figure S5 illustrate distribution of control variables and social network fragmentation, and their correlation.

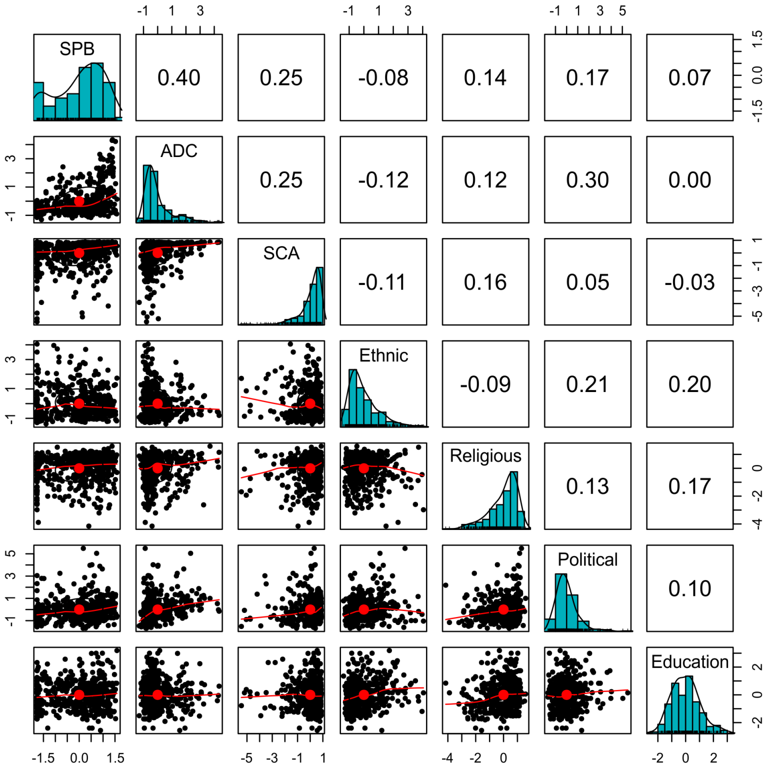

Supplementary Figure 5: Correlation and distribution of the variables in the Random Forest exercise. All the variables are standardized into z-scores.

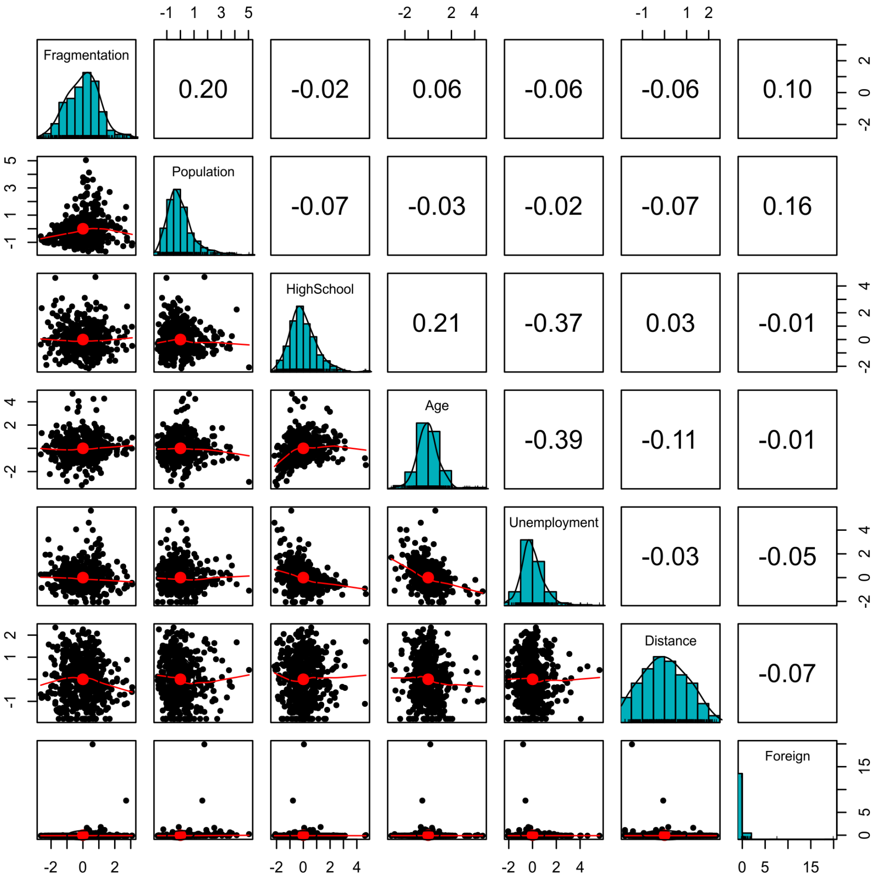

Supplementary Figure 6: Correlation and distribution of the variables in the second stage of the 2SLS regression. All the variables are standardized into z-scores.

Supplementary Note 7: The importance of urban structure in network fragmentation compared to other dimensions of segregation

We apply a Random Forest technique to rank the drivers of social network fragmentation in towns. We estimate F_i by randomly combining urban topology indicators and alternative determinants of fragmentation in 500 regressions based on decision trees. To predict variable importance we take a random sample from the decision trees and calculate the mean squared error (MSE) of the predictions applying the formula $\sum_1^e \frac{(F_i - \hat{F}_i)^2}{e}$. To quantify the importance of each determinant, we let the value of the variable in focus randomly shuffle around its mean while keeping other variables in the regression fixed and re-calculate MSE. Applying this technique informs us about the importance of observed values of the variables in focus compared to a randomized distribution. Results illustrated in Figure 7 confirm that every aspect of urban structure outperforms the alternative drivers in predicting network fragmentation.

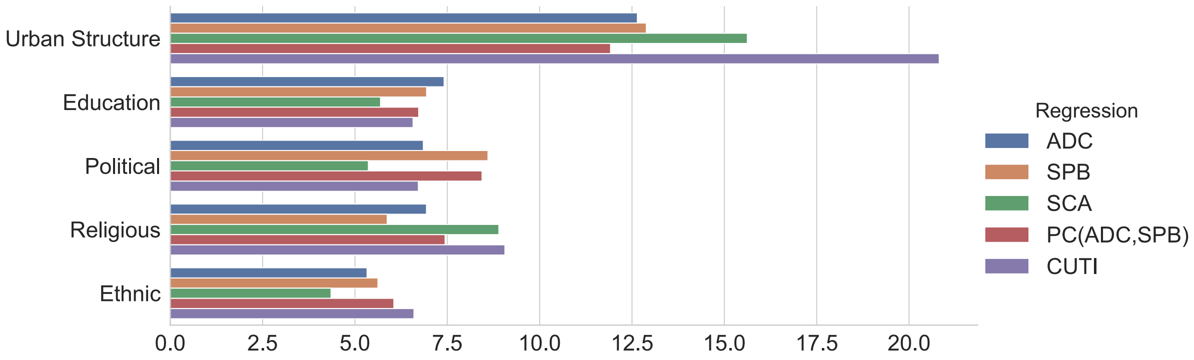

Supplementary Figure 7: **Variable importance from the Random Forest prediction of social network fragmentation in towns.** Regardless of taking different approaches, urban topology outperforms other determinants of social segregation.

Supplementary Note 8: Full tables of the 2SLS models

Supplementary Table 3: Inequality estimates, 2SLS regression, second stage. Standard errors in parentheses; all variables have been standardized.

	Dependent variable: $Gini_{2016}$				
	Instrumental Variable				
	SPB	*ADC*	*SCA*	*PC(ADC,SPB)*	*Composite Urban Topology Index*
Estimated Fragmentation	0.288**	0.408***	0.533***	0.338**	0.428***
	(0.138)	(0.153)	(0.146)	(0.138)	(0.119)
Population density	−0.067	−0.092*	−0.118**	−0.077	−0.096**
	(0.053)	(0.055)	(0.052)	(0.052)	(0.048)
High school	0.001	0.001	0.001	0.001	0.001
	(0.004)	(0.004)	(0.004)	(0.004)	(0.004)
Age	0.687	0.582	0.473	0.643	0.564
	(0.824)	(0.833)	(0.854)	(0.826)	(0.834)
Unemployment ratio	−1.221	−1.043	−0.856	−1.147	−1.012
	(1.558)	(1.575)	(1.614)	(1.561)	(1.576)
Distance to border	−0.254***	−0.243***	−0.232***	−0.249***	−0.241***
	(0.058)	(0.059)	(0.060)	(0.058)	(0.062)
Foreign investment	0.075*	0.070	0.064	0.073*	0.069
	(0.044)	(0.045)	(0.046)	(0.044)	(0.045)
Δ Foreign investment$_{2011-2016}$	−0.009	−0.012	−0.015	−0.010	−0.013
	(0.016)	(0.017)	(0.017)	(0.016)	(0.016)
Constant	−0.380	−0.386	−0.392	−0.382	−0.387
	(0.369)	(0.372)	(0.330)	(0.370)	(0.373)
County FE	Yes	Yes	Yes	Yes	Yes
First stage F-test	26.754***	22.290***	24.009***	9.635***	33.991***
Wu-Hausman tests	0.011	1.107	3.729	0.275	1.848
Sargan tests	1.400	0.051	5.349*	0.373	0.136
Observations	473	473	473	473	473
R^2	0.245	0.231	0.192	0.242	0.226
Adjusted R^2	0.200	0.186	0.145	0.197	0.181
Residual Std. Error (df = 446)	0.894	0.902	0.924	0.896	0.905

Note: $^{*}p < 0.1$; $^{**}p < 0.05$; $^{***}p < 0.01$

Supplementary Note 9: Robustness tables of the 2SLS models

Supplementary Table 4: Population Robustness with ADC in the First-Stage. Standard errors in parentheses; all variables have been standardized.

	Dependent Variable: $Gini_{2016}$		
First-stage estimator:	ADC	ADC	ADC
Population:	Main model	> 3000	> 5000
Estimated Fragmentation	0.408***	0.510**	1.033*
	(0.153)	(0.211)	(0.601)
Population density	-0.092*	-0.090	-0.111
	(0.055)	(0.057)	(0.073)
Constant	-0.386	-0.154	0.102
	(0.372)	(0.396)	(0.584)
County FE	Yes	Yes	Yes
Controls	Yes	Yes	Yes
First stage F-test	22.290***	12.546***	3.435*
Wu-Hausman tests	1.107	1.507	2.172
Sargan tests	0.051	0.033	0.327
Observations	473	420	266
Residual Std. Error	0.902 (df = 446)	0.907 (df = 393)	0.976 (df = 239)
Note:			*p<0.1; **p<0.05; ***p<0.01

Supplementary Table 5: Population Robustness with SPB in the First-Stage. Standard errors in parentheses; all variables have been standardized.

	Dependent Variable: $Gini_{2016}$		
First-stage estimator:	SPB	SPB	SPB
Population:	Main model	> 3000	> 5000
Estimated Fragmentation	0.288**	0.403**	0.568
	(0.138)	(0.197)	(0.435)
Population density	-0.067	-0.073	-0.079
	(0.053)	(0.055)	(0.062)
Constant	-0.380	-0.150	0.210
	(0.369)	(0.390)	(0.515)
County FE	Yes	Yes	Yes
Controls	Yes	Yes	Yes
First stage F-test	26.754***	13.666***	5.196**
Wu-Hausman tests	0.011	0.344	0.182
Sargan tests	1.400	1.128	2.326
Observations	473	420	266
Residual Std. Error	0.894 (df = 446)	0.893 (df = 393)	0.868 (df = 239)
Note:			*p<0.1; **p<0.05; ***p<0.01

Supplementary Table 6: Population Robustness with SCA in the First-Stage. Standard errors in parentheses; all variables have been standardized.

	Dependent Variable: Gini$_{2016}$		
First-stage estimator:	SCA	SCA	SCA
Population:	Main model	> 3000	> 5000
Estimated Fragmentation	0.534***	0.735***	1.720**
	(0.160)	(0.212)	(0.705)
Population density	-0.118**	-0.125**	-0.157*
	(0.057)	(0.060)	(0.093)
Constant	-0.393	-0.163	-0.057
	(0.382)	(0.421)	(0.763)
County FE	Yes	Yes	Yes
Controls	Yes	Yes	Yes
First stage F-test	24.009***	16.692***	3.362*
Wu-Hausman tests	3.729	6.805**	7.998**
Sargan tests	5.349*	2.585	0.614
Observations	473	420	266
Residual Std. Error	0.925 (df = 446)	0.964 (df = 393)	1.281 (df = 239)
Note:			*p<0.1; **p<0.05; ***p<0.01

Supplementary Note 10: Robustness tables of Comprehensive models to explain Inequality

Supplementary Table 7: Comprehensive model to explain Inequality. Standard errors in parentheses; all variables have been standardized.

	Dependent variable:
	$Gini_{2016}$
Fragmentation	0.197***
	(0.050)
SPB	-0.010
	(0.051)
SCA	0.106*
	(0.046)
ADC	-0.064
	(0.058)
Distance to any border	-0.259***
	(0.055)
log(Population)	0.303***
	(0.083)
log(Rail length in the town)	-0.187**
	(0.063)
Town has rail	1.438*
	(0.589)
Constant	-2.891***
	(0.767)
Observations	473
R^2	0.276
Adjusted R^2	0.234
Residual Std. Error	0.875 (df = 446)
F Statistic	6.534*** (df = 26; 446)
Note:	*p<0.05; **p<0.01; ***p<0.001

Supplementary Table 8: First-stage with all IV-s together. Standard errors in parentheses; all variables have been standardized.

	Dependent variable:
	Fragmentation
ADC	0.033
	(0.047)
SCA	0.086
	(0.046)
SPB	0.146**
	(0.047)
User rate	3.429***
	(0.544)
Constant	-1.155***
	(0.188)
Observations	473
R^2	0.192
Adjusted R^2	0.185
Residual Std. Error	0.903
F Statistic	27.831***
Note:	*p<0.05; **p<0.01; ***p<0.001

Supplementary Table 9: Second-stage with all IV-s together. Standard errors in parentheses; all variables have been standardized.

	Dependent variable:
	$Gini_{2016}$
Estimated Fragmentation	0.396**
	(0.133)
Population density	-0.089
	(0.053)
High school	0.001
	(0.004)
Age	0.592
	(0.830)
Unemployment ratio	-1.060
	(1.568)
Distance to border	-0.244***
	(0.058)
Foreign investment	0.070
	(0.044)
Δ Foreign investment	-0.012
	(0.016)
Constant	-0.385
	(0.372)
County FE	Yes
Observations	473
R^2	0.233
Adjusted R^2	0.188
Residual Std. Error	0.901 (df = 446)
Note:	*p<0.05; **p<0.01; ***p<0.001

Supplementary Note 11: 2SLS Falsification tests

Supplementary Table 10: Testing SPB as an Instrument. Standard errors in parentheses; all variables have been standardized.

	Dependent variable:			
	Fragmentation	Town Size (km^2)	Ethnic fractionalization	Roma share
	(1)	(2)	(3)	(4)
SPB	0.117*	-4.693	-0.101	-0.001
	(0.047)	(2.432)	(0.052)	(0.002)
Distance to border	-0.088	9.518***	-0.311***	-0.005*
	(0.051)	(2.682)	(0.057)	(0.002)
log(Population)	0.529***	37.378***	-0.127	-0.009***
	(0.062)	(3.209)	(0.069)	(0.003)
log(Rail length in the town)	-0.160**	11.715***	0.010	0.002
	(0.058)	(3.043)	(0.065)	(0.003)
Town has rail	1.485**	-113.375***	0.063	-0.004
	(0.548)	(28.543)	(0.611)	(0.025)
Constant	-4.444***	-277.729***	1.881**	0.095***
	(0.589)	(30.712)	(0.657)	(0.027)
County FE	Yes	Yes	Yes	Yes
Observations	473	473	473	473
R^2	0.356	0.597	0.199	0.325
Adjusted R^2	0.323	0.577	0.157	0.290
Residual Std. Error (df = 449)	0.823	42.894	0.918	0.038
F Statistic (df = 23; 449)	10.789***	28.973***	4.836***	9.399***

Note: *p<0.05; **p<0.01; ***p<0.001

Supplementary Table 11: Testing SPB as an Instrument. Standard errors in parentheses; all variables have been standardized.

	Dependent variable:							
	High School	Unemployed	Employment in Manufacturing	Doctors	Tourist Nights	Business Tax	Foreign Investment	Over 60
	(1)	(2)	(3)	(4)	(5)	(6)	(7)	(8)
SPB	0.489	-0.002	-0.003	0.005	0.0003	-0.044	-0.023	0.0001
	(0.672)	(0.002)	(0.002)	(0.007)	(0.0002)	(0.048)	(0.054)	(0.003)
Distance to border	-0.168	-0.0004	0.011***	0.008	0.0004	-0.036	-0.032	-0.008*
	(0.741)	(0.002)	(0.002)	(0.008)	(0.0002)	(0.053)	(0.060)	(0.004)
log(Population)	0.235	-0.004	0.002	-0.044***	0.0004	1.382***	0.236**	0.004
	(0.887)	(0.002)	(0.002)	(0.009)	(0.0002)	(0.064)	(0.072)	(0.004)
log(Rail length in the town)	-0.303	0.003	-0.0004	0.024**	-0.00003	0.139*	0.067	-0.008*
	(0.841)	(0.002)	(0.002)	(0.009)	(0.0002)	(0.061)	(0.068)	(0.004)
Town has rail	2.408	-0.019	0.006	-0.221**	0.0004	-1.153*	-0.719	0.076*
	(7.887)	(0.021)	(0.020)	(0.080)	(0.002)	(0.569)	(0.637)	(0.038)
Constant	21.128*	0.105***	0.092***	0.931***	0.004	-0.566	-2.169**	0.171***
	(8.486)	(0.022)	(0.021)	(0.086)	(0.002)	(0.612)	(0.685)	(0.041)
County FE	Yes	Yes	Yes	Yes	Yes	Yes	Yes	Yes
Observations	473	473	473	473	473	473	473	473
R^2	0.041	0.048	0.446	0.205	0.160	0.722	0.130	0.068
Adjusted R^2	-0.008	-0.0004	0.418	0.164	0.117	0.707	0.085	0.020
Residual Std. Error (df = 449)	11.853	0.031	0.030	0.120	0.003	0.855	0.957	0.057
F Statistic (df = 23; 449)	0.833	0.993	15.730***	5.022***	3.715***	50.607***	2.906***	1.420

Note: *p<0.05; **p<0.01; ***p<0.001

Supplementary Table 12: Testing ADC as an Instrument. Standard errors in parentheses; all variables have been standardized.

	Dependent variable:			
	Fragmentation	Town Size (km^2)	Ethnic fractionalization	Roma share
	(1)	(2)	(3)	(4)
ADC	-0.060	12.390***	-0.073	-0.001
	(0.054)	(2.728)	(0.060)	(0.002)
Distance to border	-0.094	9.381***	-0.301***	-0.005
	(0.052)	(2.630)	(0.057)	(0.002)
log(Population)	0.602***	27.357***	-0.100	-0.009**
	(0.072)	(3.656)	(0.080)	(0.003)
log(Rail length in the town)	-0.132*	9.481**	-0.001	0.002
	(0.058)	(2.969)	(0.065)	(0.003)
Town has rail	1.295*	-94.494***	0.085	-0.003
	(0.552)	(28.086)	(0.614)	(0.025)
Constant	-5.199***	-182.972***	1.718*	0.095**
	(0.671)	(34.148)	(0.746)	(0.031)
County FE	Yes	Yes	Yes	Yes
Observations	473	473	473	473
R^2	0.349	0.612	0.195	0.324
Adjusted R^2	0.315	0.592	0.153	0.290
Residual Std. Error (df = 449)	0.827	42.115	0.920	0.038
F Statistic (df = 23; 449)	10.452***	30.783***	4.716***	9.372***

Note: *p<0.05; **p<0.01; ***p<0.001

Supplementary Table 13: Testing ADC as an Instrument. Standard errors in parentheses; all variables have been standardized.

	Dependent variable:							
	High School	Unemployed	Employment in Manufacturing	Doctors	Tourist Nights	Business Tax	Foreign Investment	Over 60
	(1)	(2)	(3)	(4)	(5)	(6)	(7)	(8)
ADC	1.882*	-0.003	0.001	0.009	0.0001	-0.095	-0.028	-0.0001
	(0.763)	(0.002)	(0.002)	(0.008)	(0.0002)	(0.055)	(0.062)	(0.004)
Distance to border	-0.277	-0.0002	0.011***	0.008	0.0003	-0.029	-0.029	-0.008*
	(0.736)	(0.002)	(0.002)	(0.008)	(0.0002)	(0.053)	(0.060)	(0.004)
log(Population)	-0.987	-0.003	0.0003	-0.049***	0.0004	1.439***	0.250**	0.004
	(1.023)	(0.003)	(0.003)	(0.010)	(0.0003)	(0.074)	(0.083)	(0.005)
log(Rail length in the town)	-0.418	0.003	-0.001	0.024**	0.00003	0.141*	0.066	-0.008*
	(0.831)	(0.002)	(0.002)	(0.008)	(0.0002)	(0.060)	(0.067)	(0.004)
Town has rail	4.032	-0.020	0.010	-0.216**	0.0001	-1.215*	-0.726	0.076*
	(7.856)	(0.021)	(0.020)	(0.080)	(0.002)	(0.569)	(0.638)	(0.038)
Constant	31.788***	0.095***	0.109***	0.972***	0.003	-1.045	-2.277**	0.170***
	(9.552)	(0.025)	(0.024)	(0.098)	(0.003)	(0.692)	(0.776)	(0.046)
County FE	Yes	Yes	Yes	Yes	Yes	Yes	Yes	Yes
Observations	473	473	473	473	473	473	473	473
R^2	0.053	0.049	0.444	0.206	0.154	0.723	0.130	0.068
Adjusted R^2	0.004	0.0003	0.415	0.165	0.110	0.709	0.085	0.020
Residual Std. Error (df = 449)	11.780	0.031	0.030	0.120	0.003	0.853	0.957	0.057
F Statistic (df = 23; 449)	1.084	1.006	15.585***	5.059***	3.549***	50.940***	2.907***	1.420
Note:								*p<0.05; **p<0.01; ***p<0.001

Supplementary Table 14: Testing SCA as an Instrument. Standard errors in parentheses; all variables have been standardized.

	Dependent variable:			
	Fragmentation	Town Size (km^2)	Ethnic fractionalization	Roma share
	(1)	(2)	(3)	(4)
SCA	0.064	0.968	-0.104*	-0.007***
	(0.043)	(2.237)	(0.048)	(0.002)
Distance to border	-0.094	9.897***	-0.308***	-0.005*
	(0.052)	(2.688)	(0.057)	(0.002)
log(Population)	0.533***	35.826***	-0.112	-0.007*
	(0.063)	(3.283)	(0.070)	(0.003)
log(Rail length in the town)	-0.140*	10.806***	-0.005	0.002
	(0.058)	(3.022)	(0.064)	(0.003)
Town has rail	1.372*	-108.389***	0.154	-0.003
	(0.548)	(28.545)	(0.608)	(0.025)
Constant	-4.611***	-260.182***	1.873**	0.076**
	(0.589)	(30.668)	(0.653)	(0.027)
Observations	473	473	473	473
R^2	0.350	0.594	0.200	0.345
Adjusted R^2	0.317	0.573	0.159	0.312
Residual Std. Error (df = 449)	0.827	43.062	0.917	0.037
F Statistic (df = 23; 449)	10.517***	28.594***	4.892***	10.298***
Note:				*p<0.05; **p<0.01; ***p<0.001

Supplementary Table 15: Testing SCA as an Instrument. Standard errors in parentheses; all variables have been standardized.

	Dependent variable:							
	High School	Unemployed	Employment in Manufacturing	Doctors	Tourist Nights	Business Tax	Foreign Investment	Over 60
	(1)	(2)	(3)	(4)	(5)	(6)	(7)	(8)
SCA	0.764	0.002	-0.001	0.013*	0.001***	0.169***	-0.031	0.003
	(0.615)	(0.002)	(0.002)	(0.006)	(0.0002)	(0.044)	(0.050)	(0.003)
Distance to border	-0.176	-0.0002	0.011***	0.008	0.0004	-0.026	-0.031	-0.008*
	(0.739)	(0.002)	(0.002)	(0.007)	(0.0002)	(0.053)	(0.060)	(0.004)
log(Population)	0.057	-0.006*	0.002	-0.048***	0.0002	1.304***	0.242***	0.003
	(0.903)	(0.002)	(0.002)	(0.009)	(0.0002)	(0.064)	(0.073)	(0.004)
log(Rail length in the town)	-0.238	0.002	-0.001	0.025**	0.00001	0.125*	0.064	-0.008*
	(0.831)	(0.002)	(0.002)	(0.008)	(0.0002)	(0.059)	(0.067)	(0.004)
Town has rail	2.004	-0.017	0.008	-0.224**	0.0001	-1.084	-0.699	0.077*
	(7.848)	(0.021)	(0.020)	(0.079)	(0.002)	(0.558)	(0.634)	(0.037)
Constant	21.957**	0.118***	0.096***	0.955***	0.005*	0.093	-2.192**	0.181***
	(8.431)	(0.022)	(0.021)	(0.085)	(0.002)	(0.600)	(0.681)	(0.040)
County FE	Yes	Yes	Yes	Yes	Yes	Yes	Yes	Yes
Observations	473	473	473	473	473	473	473	473
R^2	0.043	0.049	0.444	0.212	0.195	0.730	0.130	0.070
Adjusted R^2	-0.006	-0.0001	0.416	0.171	0.153	0.716	0.085	0.023
Residual Std. Error (df = 449)	11.839	0.031	0.030	0.120	0.003	0.842	0.956	0.057
F Statistic (df = 23; 449)	0.879	0.998	15.592***	5.238***	4.717***	52.812***	2.916***	1.479

Note: *p<0.05; **p<0.01; ***p<0.001

References

[1] B. Lengyel, Á. Jakobi, Online social networks, location, and the dual effect of distance from the centre, Tijdschrift voor economische en sociale geografie **107**(3), 298 (2016)
