# Supplementary material for: Inequality is rising where social network segregation interacts with urban topology
Source: Nat Commun. 2021 Feb 18;12:1143. doi: 10.1038/s41467-021-21465-0 (PMC7892860; doi:10.1038/s41467-021-21465-0)
Supplement: Supplementary file 2 — Reporting Summary [file 41467_2021_21465_MOESM2_ESM.pdf]

nature research

Corresponding author(s): Balázs Lengyel

Last updated by author(s): Jan 19, 2021

Reporting Summary

Nature Research wishes to improve the reproducibility of the work that we publish. This form provides structure for consistency and transparency in reporting. For further information on Nature Research policies, see our Editorial Policies and the Editorial Policy Checklist.

Statistics

For all statistical analyses, confirm that the following items are present in the figure legend, table legend, main text, or Methods section.

n/a	Confirmed	
☐	☒	The exact sample size (n) for each experimental group/condition, given as a discrete number and unit of measurement
☒	☐	A statement on whether measurements were taken from distinct samples or whether the same sample was measured repeatedly
☐	☒	The statistical test(s) used AND whether they are one- or two-sided<br>*Only common tests should be described solely by name; describe more complex techniques in the Methods section.*
☐	☒	A description of all covariates tested
☐	☒	A description of any assumptions or corrections, such as tests of normality and adjustment for multiple comparisons
☐	☒	A full description of the statistical parameters including central tendency (e.g. means) or other basic estimates (e.g. regression coefficient) AND variation (e.g. standard deviation) or associated estimates of uncertainty (e.g. confidence intervals)
☐	☒	For null hypothesis testing, the test statistic (e.g. F, t, r) with confidence intervals, effect sizes, degrees of freedom and P value noted<br>*Give P values as exact values whenever suitable.*
☒	☐	For Bayesian analysis, information on the choice of priors and Markov chain Monte Carlo settings
☒	☐	For hierarchical and complex designs, identification of the appropriate level for tests and full reporting of outcomes
☐	☒	Estimates of effect sizes (e.g. Cohen's d, Pearson's r), indicating how they were calculated

Our web collection on statistics for biologists contains articles on many of the points above.

Software and code

Policy information about availability of computer code

Data collection	We use Python (version number 3.7.2) scripts to collect OpenStreetMap, Point of Interest data and to process Corine Land Cover data. Code and data has been reposited at https://zenodo.org/record/4448183#.YAXjOOhKg2w.
Data analysis	R (version number 4.0.0) and Python (version number 3.7.2) scripts are used for data analysis. R algorithms: Louvain community detection algorithm (igraph package, version 1.2.4), Random Forest regression (randomForest package, version 4.6-14), Instrumental-variable regression (AER package, version 1.2-9). Python algorithms: DBSCAN (scikit-learn package, version 0.21). Code and data has been reposited at https://zenodo.org/record/4448183#.YAXjOOhKg2w.

For manuscripts utilizing custom algorithms or software that are central to the research but not yet described in published literature, software must be made available to editors and reviewers. We strongly encourage code deposition in a community repository (e.g. GitHub). See the Nature Research guidelines for submitting code & software for further information.

Data

Policy information about availability of data

All manuscripts must include a data availability statement. This statement should provide the following information, where applicable:

- Accession codes, unique identifiers, or web links for publicly available datasets
- A list of figures that have associated raw data
- A description of any restrictions on data availability

Data tenure was controlled by a non-disclosure agreement between the owner of iWiW data and the research group. Raw data are not publicly available due to privacy considerations, but are available to researchers who meet the criteria for access to confidential data, sign a confidentiality agreement and agree to work under supervision at the Centre for Economic- and Regional Studies. The table that contains town-level variables, can be accessed at https://zenodo.org/record/4448183#.YAXjOOhKg2w. Town-level aggregate socio-economic information can be accessed at https://www.teir.hu. The Corine Land Cover (CLC) data is

available at https://land.copernicus.eu/pan-european/corine-land-cover website. The Open Street Map data is accessible at https://data2.openstreetmap.hu/hatarok/ and the POI can be downloaded from https://download.geofabrik.de/.

Field-specific reporting

Please select the one below that is the best fit for your research. If you are not sure, read the appropriate sections before making your selection.

- [ ] Life sciences
- [x] Behavioural & social sciences
- [ ] Ecological, evolutionary & environmental sciences

For a reference copy of the document with all sections, see nature.com/documents/nr-reporting-summary-flat.pdf

Behavioural & social sciences study design

All studies must disclose on these points even when the disclosure is negative.

Study description	This is a quantitative study applying network science techniques, GIS tools, random forest models and linear and IV regressions.
Research sample	The research sample is social networks of individuals resided in Hungarian towns that have more than 2,500 population. The capital Budapest is excluded from our sample.
Sampling strategy	We exclude capital city Budapest from the analysis because it is a unique settlement in several ways. It contains twenty administrative subunits which serve as weak social and political barriers - we do not observe this granularity in our social network data. It is also an order of magnitude outlier in population, density, and physical size and would introduce significant leverage in our regression models.<br>Towns smaller than 2,500 population were excluded because the penetration of iWiW users were very low in these locations. Such low representativity would potentially bias the social network indicators.
Data collection	iWiW data were collected by the data owner company. The data shared with us contains individual information that were publicly available on the website. All observations were anonymized.<br>Settlement-level socio-economic indicators and urban topology information were collected by the authors.
Timing	iWiW data were collected by the data owner from January 14 2013 until January 25 2013. Open-access urban information including residential area and physical barrier polygons were collected from April 2 2018 until July 27 2018.
Data exclusions	We excluded those profiles that collected more than 5000 online connections and those that the registration date and last log-in date fell on the same day. The prior set of users are celebrities, public figures, or sometimes commercial agents whose online connections do not represent acquaintance-based social relations that our analysis requires. The latter profiles have been exclusively created for sending out spam messages.
Non-participation	The iwiw data represents 50% of the population who have been using the internet. Underrepresented groups include the poor who are thought to be most segregated from social interaction in towns. Therefore our results might underestimate the role of social network fragmentation in income inequality dynamics.
Randomization	We apply a Random Forest technique to rank the drivers of social network fragmentation in towns. We estimate social network fragmentation by randomly combining urban topology indicators and alternative determinants of fragmentation in 500 regressions based on decision trees. To predict variable importance we take a random sample from the decision trees and calculate the mean squared error of the predictions. To quantify the importance of each determinant, we let the value of the variable in focus randomly shuffle around its mean while keeping other variables in the regression fixed. Applying this technique informs us about the importance of observed values of the variables in focus compared to a randomized distribution.

Reporting for specific materials, systems and methods

We require information from authors about some types of materials, experimental systems and methods used in many studies. Here, indicate whether each material, system or method listed is relevant to your study. If you are not sure if a list item applies to your research, read the appropriate section before selecting a response.

Materials & experimental systems

n/a	Involved in the study	
x		Antibodies
x		Eukaryotic cell lines
x		Palaeontology and archaeology
x		Animals and other organisms
x		Human research participants
x		Clinical data
x		Dual use research of concern

Methods

n/a	Involved in the study	
x		ChIP-seq
x		Flow cytometry
x		MRI-based neuroimaging
